# *rac*-Phenyl (benzylamido)(*p*-tolyl­amido)­phosphinate

**DOI:** 10.1107/S1600536811034465

**Published:** 2011-08-31

**Authors:** Mehrdad Pourayoubi, Fatemeh Karimi Ahmadabad, Marek Nečas

**Affiliations:** a Department of Chemistry, Ferdowsi University of Mashhad, Mashhad 91779, Iran; b Department of Chemistry, Faculty of Science, Masaryk University, Kotlarska 2, Brno CZ-61137, Czech Republic

## Abstract

The title compound, C_20_H_21_N_2_O_2_P, was synthesized from (*RS*)-(C_6_H_5_O)P(O)Cl(NHC_6_H_4_-*p*-CH_3_) and benzyl­amine. The product crystallizes as a racemate in a polar space group. The phospho­rus atom has a distorted tetra­hedral configuration: the bond angles at the P atom are in the range 103.2 (1)–118.4 (1)°. The P—N(benzyl­amido) bond [1.615 (2) Å] is slightly shorter than the P—N(*p*-tolyl­amido) bond [1.630 (2) Å]. Both N—H groups adopt an *anti* orientation relative to the phosphoryl group. In the crystal, the adjacent mol­ecules are linked *via* N—H⋯O hydrogen bonds, forming *R*_2_^2^(8) rings, into a one-dimensional arrangement parallel to the *x* axis.

## Related literature

For a related mixed-amido phosphinate derivative and its mol­ecular geometry, see: Sabbaghi *et al.* (2011 ▶). For graph-set notation, see: Bernstein *et al.* (1995 ▶).
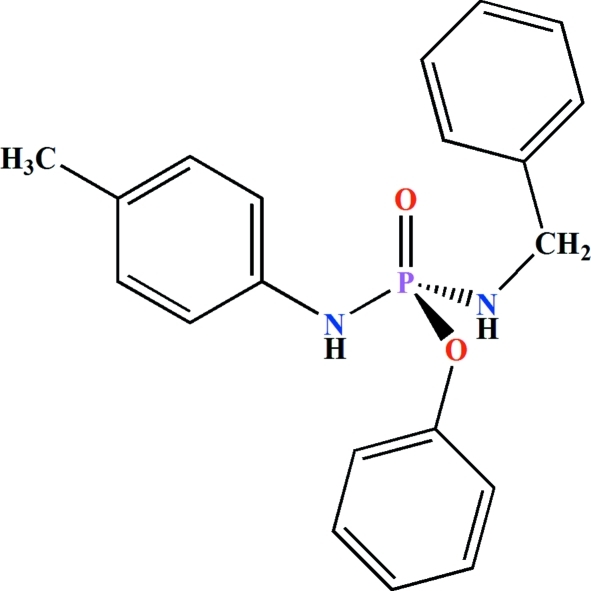


## Experimental

### 

#### Crystal data

C_20_H_21_N_2_O_2_P
*M* *_r_* = 352.36
Orthorhombic, 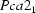

*a* = 9.6986 (5) Å
*b* = 13.0751 (6) Å
*c* = 14.3446 (5) Å
*V* = 1819.04 (14) Å^3^
*Z* = 4
Mo *K*α radiation
μ = 0.17 mm^−1^
*T* = 120 K
0.30 × 0.30 × 0.10 mm

#### Data collection

Xcalibur, Sapphire2, large Be window diffractometer
Absorption correction: multi-scan (*CrysAlis PRO*; Oxford Diffraction, 2009 ▶) *T*_min_ = 0.785, *T*_max_ = 1.000
20413 measured reflections
3200 independent reflections
2823 reflections with *I* > 2σ(*I*)
*R*_int_ = 0.047

#### Refinement

*R*[*F* ^2^ > 2σ(*F* ^2^)] = 0.041
*wR*(*F* ^2^) = 0.099
*S* = 1.00
3200 reflections
233 parameters
1 restraint
H atoms treated by a mixture of independent and constrained refinement
Δρ_max_ = 0.51 e Å^−3^
Δρ_min_ = −0.29 e Å^−3^
Absolute structure: Flack (1983 ▶), 1526 Friedel pairs
Flack parameter: 0.01 (10)

### 

Data collection: *CrysAlis PRO* (Oxford Diffraction, 2009 ▶); cell refinement: *CrysAlis PRO*; data reduction: *CrysAlis PRO*; program(s) used to solve structure: *SHELXS97* (Sheldrick, 2008 ▶); program(s) used to refine structure: *SHELXL97* (Sheldrick, 2008 ▶); molecular graphics: *Mercury* (Macrae *et al.*, 2008 ▶); software used to prepare material for publication: *enCIFer* (Allen *et al.*, 2004 ▶).

## Supplementary Material

Crystal structure: contains datablock(s) I, global. DOI: 10.1107/S1600536811034465/ld2023sup1.cif

Structure factors: contains datablock(s) I. DOI: 10.1107/S1600536811034465/ld2023Isup2.hkl

Additional supplementary materials:  crystallographic information; 3D view; checkCIF report

## Figures and Tables

**Table 1:** Hydrogen-bond geometry (Å, °)

*D*—H⋯*A*	*D*—H	H⋯*A*	*D*⋯*A*	*D*—H⋯*A*
N1—H1*N*⋯O1^i^	0.76 (3)	2.43 (3)	3.127 (3)	153 (3)
N2—H2*N*⋯O2^i^	0.86 (3)	1.91 (3)	2.761 (3)	176 (3)

Symmetry code: (i) 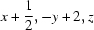.

**Fig. 1.**
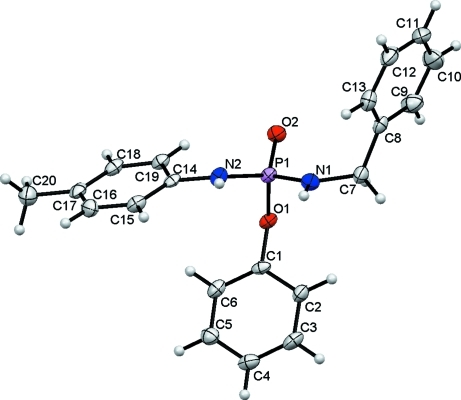
An ORTEP style plot and atom labeling scheme for the title compound. Displacement ellipsoids are given at 50% probability level and H atoms are drawn as small spheres of an arbitrary radius.

**Fig. 2.**
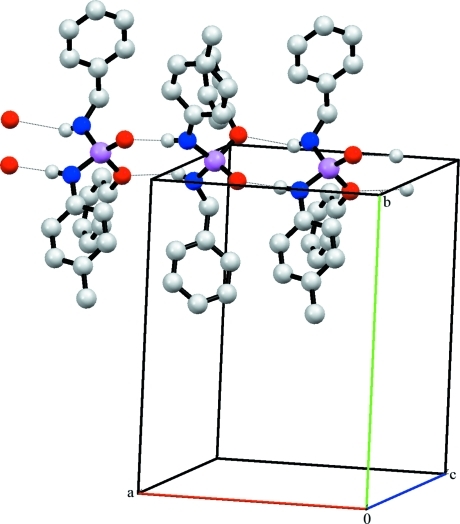
Part of the crystal packing of the title compound with the hydrogen bonds shown as dotted lines (the C—H hydrogen atoms are omitted for clarity).

## supplementary crystallographic information

### Comment

In continuation of the previous works on synthesis and structure determination of mixed-amido phosphinates with common formula (RO)(N*R*^1^*R*^2^)(N*R*^3^*R*^4^)P(O) (Sabbaghi *et al.*, 2011; and the related reference cited therein), the structure of the title molecule, [C_6_H_5_O][4-CH_3_C_6_H_4_NH][C_6_H_5_CH_2_NH]PO (Fig. 1), is reported here.

Single crystals were obtained from CHCl_3_/CH_3_CN at room temperature.

The P═O (1.4679 (17) Å), P—O (1.6250 (17) Å), P—N (1.615 (2) Å & 1.630 (2) Å) and C—O (1.400 (3) Å) bond lengths and the P—N—C (120.54 (18)° & 125.18 (18)°) and P—O—C (120.90 (14)°) bond angles are within the expected values (Sabbaghi *et al.*, 2011).

The phosphorus atom has a distorted tetrahedral P(═O)(O)(N)(N) environment. The bond angles at the P atom are in the range from 103.17 (9)° [for the O2—P1—O1 angle] to 118.39 (11)° [for the O2—P1—N2 angle].

In the crystal structure, neighbouring molecules are H-bonded *via* N—H···O(P) hydrogen bonds, building *R*_2_^2^(8) rings (Bernstein *et al.*, 1995), in a linear arrangement parallel to [100], Table 1, Fig. 2.

### Experimental

To a solution of (C_6_H_5_O)(4-CH_3_C_6_H_4_NH)P(O)Cl (2.286 mmol) in chloroform, a solution of benzylamine (4.572 mmol) in chloroform was added at 273 K. After stirring for 5 h, the solvent was removed and the obtained solid was washed with distilled water. Single crystals were obtained from a solution of the title compound in CH_3_CN/CHCl_3_ after slow evaporation at room temperature.

### Refinement

All carbon-bound H atoms were placed in calculated positions and were refined as riding with their *U*_iso_ set to be either 1.2*U*_eq_ or 1.5*U*_eq_ (methyl) of the respective carrier atoms; in addition, the methyl H atoms were allowed to rotate about the C—C bond. Nitrogen-bound H atoms were located in a difference Fourier map and refined with their *U*_iso_ set to 1.2*U*_eq_ of the adjacent nitrogen atoms.

### Crystal data

**Table:**

C_20_H_21_N_2_O_2_P	*D*_x_ = 1.287 Mg m^−^^3^
*M**_r_* = 352.36	Mo *K*α radiation, λ = 0.71073 Å
Orthorhombic, *P**c**a*2_1_	Cell parameters from 10547 reflections
*a* = 9.6986 (5) Å	θ = 3.0–27.2°
*b* = 13.0751 (6) Å	µ = 0.17 mm^−^^1^
*c* = 14.3446 (5) Å	*T* = 120 K
*V* = 1819.04 (14) Å^3^	Plate, colorless
*Z* = 4	0.30 × 0.30 × 0.10 mm
*F*(000) = 744	

### Data collection

**Table:**

Xcalibur, Sapphire2, large Be window diffractometer	3200 independent reflections
Radiation source: Enhance (Mo) X-ray Source	2823 reflections with *I* > 2σ(*I*)
graphite	*R*_int_ = 0.047
Detector resolution: 8.4353 pixels mm^-1^	θ_max_ = 25.0°, θ_min_ = 3.0°
ω scan	*h* = −11→9
Absorption correction: multi-scan (*CrysAlis PRO*; Oxford Diffraction, 2009)	*k* = −15→15
*T*_min_ = 0.785, *T*_max_ = 1.000	*l* = −17→17
20413 measured reflections	

### Refinement

**Table:**

Refinement on *F*^2^	Secondary atom site location: difference Fourier map
Least-squares matrix: full	Hydrogen site location: inferred from neighbouring sites
*R*[*F*^2^ > 2σ(*F*^2^)] = 0.041	H atoms treated by a mixture of independent and constrained refinement
*wR*(*F*^2^) = 0.099	*w* = 1/[σ^2^(*F*_o_^2^) + (0.073*P*)^2^] where *P* = (*F*_o_^2^ + 2*F*_c_^2^)/3
*S* = 1.00	(Δ/σ)_max_ < 0.001
3200 reflections	Δρ_max_ = 0.51 e Å^−^^3^
233 parameters	Δρ_min_ = −0.29 e Å^−^^3^
1 restraint	Absolute structure: Flack (1983), 1523 Friedel pairs
Primary atom site location: structure-invariant direct methods	Flack parameter: 0.01 (10)

### Special details

**Table:**

Geometry. All e.s.d.'s (except the e.s.d. in the dihedral angle between two l.s. planes) are estimated using the full covariance matrix. The cell e.s.d.'s are taken into account individually in the estimation of e.s.d.'s in distances, angles and torsion angles; correlations between e.s.d.'s in cell parameters are only used when they are defined by crystal symmetry. An approximate (isotropic) treatment of cell e.s.d.'s is used for estimating e.s.d.'s involving l.s. planes.
Refinement. Refinement of *F*^2^ against ALL reflections. The weighted *R*-factor *wR* and goodness of fit *S* are based on *F*^2^, conventional *R*-factors *R* are based on *F*, with *F* set to zero for negative *F*^2^. The threshold expression of *F*^2^ > σ(*F*^2^) is used only for calculating *R*-factors(gt) *etc*. and is not relevant to the choice of reflections for refinement. *R*-factors based on *F*^2^ are statistically about twice as large as those based on *F*, and *R*- factors based on ALL data will be even larger.

### Fractional atomic coordinates and isotropic or equivalent isotropic displacement parameters (Å^2^)

**Table:**

	*x*	*y*	*z*	*U*_iso_*/*U*_eq_	
P1	0.91905 (5)	0.99930 (4)	0.56899 (5)	0.01847 (16)	
O1	0.84602 (16)	1.07673 (12)	0.64256 (11)	0.0217 (4)	
O2	0.80162 (17)	0.94641 (13)	0.52605 (12)	0.0247 (4)	
N1	1.0250 (2)	0.92421 (16)	0.62255 (15)	0.0241 (5)	
H1N	1.098 (3)	0.944 (2)	0.630 (2)	0.029*	
N2	1.0197 (2)	1.06729 (15)	0.50236 (15)	0.0217 (5)	
H2N	1.107 (3)	1.062 (2)	0.5071 (19)	0.026*	
C1	0.9250 (2)	1.1424 (2)	0.69810 (16)	0.0207 (6)	
C2	0.9809 (3)	1.1052 (2)	0.78124 (17)	0.0264 (6)	
H2C	0.9699	1.0356	0.7987	0.032*	
C3	1.0533 (3)	1.1733 (2)	0.83765 (18)	0.0297 (6)	
H3A	1.0940	1.1494	0.8938	0.036*	
C4	1.0667 (3)	1.2738 (2)	0.81375 (18)	0.0290 (6)	
H4A	1.1156	1.3192	0.8535	0.035*	
C5	1.0088 (3)	1.3098 (2)	0.73111 (18)	0.0283 (6)	
H5A	1.0182	1.3798	0.7143	0.034*	
C6	0.9375 (3)	1.2432 (2)	0.67366 (17)	0.0249 (6)	
H6A	0.8974	1.2674	0.6174	0.030*	
C7	0.9737 (3)	0.83908 (19)	0.67821 (18)	0.0273 (6)	
H7A	0.8801	0.8564	0.7006	0.033*	
H7B	1.0336	0.8315	0.7337	0.033*	
C8	0.9671 (3)	0.73740 (18)	0.62858 (16)	0.0225 (5)	
C9	0.8580 (3)	0.6727 (2)	0.6440 (2)	0.0360 (7)	
H9A	0.7840	0.6945	0.6826	0.043*	
C10	0.8539 (4)	0.5761 (2)	0.6042 (2)	0.0503 (9)	
H10A	0.7787	0.5316	0.6167	0.060*	
C11	0.9596 (4)	0.5448 (2)	0.54621 (18)	0.0436 (8)	
H11A	0.9573	0.4789	0.5184	0.052*	
C12	1.0667 (3)	0.6091 (2)	0.52932 (19)	0.0368 (7)	
H12A	1.1394	0.5876	0.4895	0.044*	
C13	1.0716 (3)	0.70547 (18)	0.5693 (2)	0.0282 (5)	
H13A	1.1467	0.7498	0.5561	0.034*	
C14	0.9794 (3)	1.15603 (17)	0.45273 (16)	0.0196 (5)	
C15	1.0762 (3)	1.23182 (19)	0.43674 (17)	0.0246 (5)	
H15A	1.1680	1.2239	0.4588	0.030*	
C16	1.0383 (3)	1.3201 (2)	0.38799 (19)	0.0296 (6)	
H16A	1.1061	1.3707	0.3753	0.036*	
C17	0.9042 (3)	1.33547 (18)	0.35774 (16)	0.0258 (6)	
C18	0.8104 (3)	1.25870 (19)	0.37405 (16)	0.0249 (6)	
H18A	0.7182	1.2670	0.3530	0.030*	
C19	0.8467 (3)	1.16882 (19)	0.42071 (16)	0.0229 (5)	
H19A	0.7799	1.1167	0.4303	0.027*	
C20	0.8653 (3)	1.4309 (2)	0.3061 (2)	0.0364 (7)	
H20A	0.7646	1.4369	0.3040	0.055*	
H20B	0.9017	1.4276	0.2424	0.055*	
H20C	0.9041	1.4905	0.3381	0.055*	

### Atomic displacement parameters (Å^2^)

**Table:**

	*U*^11^	*U*^22^	*U*^33^	*U*^12^	*U*^13^	*U*^23^
P1	0.0170 (3)	0.0230 (3)	0.0154 (3)	−0.0002 (3)	0.0001 (3)	0.0003 (2)
O1	0.0207 (9)	0.0288 (9)	0.0155 (8)	0.0002 (7)	−0.0003 (7)	−0.0013 (7)
O2	0.0199 (9)	0.0295 (10)	0.0247 (8)	−0.0025 (7)	−0.0001 (7)	−0.0008 (7)
N1	0.0185 (11)	0.0276 (12)	0.0261 (12)	−0.0036 (10)	−0.0027 (10)	0.0003 (9)
N2	0.0153 (11)	0.0287 (11)	0.0211 (10)	0.0021 (9)	−0.0001 (9)	0.0026 (9)
C1	0.0165 (13)	0.0302 (14)	0.0154 (12)	−0.0025 (10)	0.0021 (9)	−0.0065 (10)
C2	0.0320 (15)	0.0292 (13)	0.0180 (11)	0.0010 (11)	−0.0026 (11)	0.0026 (11)
C3	0.0307 (15)	0.0421 (16)	0.0163 (12)	0.0023 (13)	−0.0032 (11)	−0.0008 (12)
C4	0.0274 (14)	0.0378 (15)	0.0217 (13)	−0.0048 (12)	−0.0014 (11)	−0.0077 (11)
C5	0.0308 (16)	0.0282 (14)	0.0260 (15)	−0.0039 (12)	0.0044 (12)	−0.0031 (12)
C6	0.0239 (14)	0.0340 (14)	0.0167 (11)	0.0031 (11)	0.0007 (10)	0.0015 (11)
C7	0.0368 (17)	0.0260 (14)	0.0190 (13)	0.0026 (12)	−0.0008 (12)	0.0046 (11)
C8	0.0241 (13)	0.0262 (13)	0.0173 (12)	0.0033 (11)	−0.0028 (10)	0.0056 (10)
C9	0.0355 (16)	0.0454 (16)	0.0272 (14)	−0.0088 (13)	0.0049 (12)	−0.0043 (13)
C10	0.066 (2)	0.0461 (18)	0.0387 (16)	−0.0296 (17)	0.0033 (17)	−0.0012 (14)
C11	0.083 (2)	0.0280 (14)	0.0199 (14)	−0.0073 (16)	−0.0028 (14)	−0.0025 (11)
C12	0.054 (2)	0.0357 (15)	0.0201 (13)	0.0115 (14)	0.0042 (13)	0.0011 (11)
C13	0.0289 (14)	0.0316 (13)	0.0240 (12)	0.0016 (10)	0.0020 (12)	0.0081 (13)
C14	0.0214 (14)	0.0257 (13)	0.0118 (12)	0.0031 (11)	0.0044 (10)	−0.0037 (10)
C15	0.0229 (14)	0.0327 (13)	0.0184 (12)	0.0010 (11)	−0.0016 (10)	−0.0015 (11)
C16	0.0361 (17)	0.0274 (13)	0.0254 (13)	−0.0051 (12)	0.0057 (12)	−0.0009 (11)
C17	0.0368 (16)	0.0279 (14)	0.0128 (12)	0.0071 (11)	0.0029 (10)	−0.0027 (10)
C18	0.0252 (14)	0.0341 (14)	0.0154 (12)	0.0058 (11)	0.0008 (10)	−0.0026 (11)
C19	0.0237 (14)	0.0295 (13)	0.0154 (11)	0.0002 (11)	0.0010 (10)	−0.0004 (10)
C20	0.0481 (18)	0.0310 (14)	0.0302 (14)	0.0037 (14)	0.0008 (13)	0.0023 (12)

### Geometric parameters (Å, °)

**Table:**

P1—O2	1.4679 (17)	C8—C13	1.387 (4)
P1—N1	1.615 (2)	C9—C10	1.386 (4)
P1—O1	1.6250 (17)	C9—H9A	0.9500
P1—N2	1.630 (2)	C10—C11	1.382 (4)
O1—C1	1.400 (3)	C10—H10A	0.9500
N1—C7	1.457 (3)	C11—C12	1.358 (4)
N1—H1N	0.76 (3)	C11—H11A	0.9500
N2—C14	1.416 (3)	C12—C13	1.385 (4)
N2—H2N	0.86 (3)	C12—H12A	0.9500
C1—C6	1.369 (4)	C13—H13A	0.9500
C1—C2	1.398 (4)	C14—C19	1.377 (4)
C2—C3	1.393 (4)	C14—C15	1.385 (3)
C2—H2C	0.9500	C15—C16	1.399 (4)
C3—C4	1.364 (4)	C15—H15A	0.9500
C3—H3A	0.9500	C16—C17	1.386 (4)
C4—C5	1.394 (4)	C16—H16A	0.9500
C4—H4A	0.9500	C17—C18	1.375 (4)
C5—C6	1.384 (4)	C17—C20	1.499 (3)
C5—H5A	0.9500	C18—C19	1.397 (4)
C6—H6A	0.9500	C18—H18A	0.9500
C7—C8	1.510 (4)	C19—H19A	0.9500
C7—H7A	0.9900	C20—H20A	0.9800
C7—H7B	0.9900	C20—H20B	0.9800
C8—C9	1.373 (4)	C20—H20C	0.9800
			
O2—P1—N1	114.00 (11)	C8—C9—C10	121.1 (3)
O2—P1—O1	103.17 (9)	C8—C9—H9A	119.4
N1—P1—O1	110.30 (11)	C10—C9—H9A	119.4
O2—P1—N2	118.39 (11)	C11—C10—C9	119.8 (3)
N1—P1—N2	103.28 (11)	C11—C10—H10A	120.1
O1—P1—N2	107.58 (10)	C9—C10—H10A	120.1
C1—O1—P1	120.90 (14)	C12—C11—C10	119.4 (3)
C7—N1—P1	120.54 (18)	C12—C11—H11A	120.3
C7—N1—H1N	120 (2)	C10—C11—H11A	120.3
P1—N1—H1N	117 (2)	C11—C12—C13	121.0 (3)
C14—N2—P1	125.18 (18)	C11—C12—H12A	119.5
C14—N2—H2N	112.1 (18)	C13—C12—H12A	119.5
P1—N2—H2N	120.5 (19)	C12—C13—C8	120.2 (2)
C6—C1—C2	121.3 (2)	C12—C13—H13A	119.9
C6—C1—O1	119.6 (2)	C8—C13—H13A	119.9
C2—C1—O1	118.9 (2)	C19—C14—C15	119.5 (2)
C3—C2—C1	117.9 (2)	C19—C14—N2	121.7 (2)
C3—C2—H2C	121.1	C15—C14—N2	118.8 (2)
C1—C2—H2C	121.1	C14—C15—C16	119.7 (2)
C4—C3—C2	121.2 (2)	C14—C15—H15A	120.2
C4—C3—H3A	119.4	C16—C15—H15A	120.2
C2—C3—H3A	119.4	C17—C16—C15	121.5 (2)
C3—C4—C5	120.1 (3)	C17—C16—H16A	119.2
C3—C4—H4A	120.0	C15—C16—H16A	119.2
C5—C4—H4A	120.0	C18—C17—C16	117.5 (2)
C6—C5—C4	119.7 (2)	C18—C17—C20	121.7 (2)
C6—C5—H5A	120.2	C16—C17—C20	120.8 (2)
C4—C5—H5A	120.2	C17—C18—C19	121.9 (2)
C1—C6—C5	119.8 (2)	C17—C18—H18A	119.0
C1—C6—H6A	120.1	C19—C18—H18A	119.0
C5—C6—H6A	120.1	C14—C19—C18	119.8 (2)
N1—C7—C8	115.4 (2)	C14—C19—H19A	120.1
N1—C7—H7A	108.4	C18—C19—H19A	120.1
C8—C7—H7A	108.4	C17—C20—H20A	109.5
N1—C7—H7B	108.4	C17—C20—H20B	109.5
C8—C7—H7B	108.4	H20A—C20—H20B	109.5
H7A—C7—H7B	107.5	C17—C20—H20C	109.5
C9—C8—C13	118.5 (2)	H20A—C20—H20C	109.5
C9—C8—C7	119.9 (2)	H20B—C20—H20C	109.5
C13—C8—C7	121.6 (2)		
			
O2—P1—O1—C1	−179.64 (16)	N1—C7—C8—C13	41.2 (3)
N1—P1—O1—C1	58.23 (19)	C13—C8—C9—C10	2.3 (4)
N2—P1—O1—C1	−53.74 (19)	C7—C8—C9—C10	−175.7 (3)
O2—P1—N1—C7	−40.2 (2)	C8—C9—C10—C11	−1.5 (5)
O1—P1—N1—C7	75.3 (2)	C9—C10—C11—C12	0.3 (5)
N2—P1—N1—C7	−169.97 (18)	C10—C11—C12—C13	0.0 (4)
O2—P1—N2—C14	64.2 (2)	C11—C12—C13—C8	0.8 (4)
N1—P1—N2—C14	−168.78 (18)	C9—C8—C13—C12	−1.9 (4)
O1—P1—N2—C14	−52.1 (2)	C7—C8—C13—C12	176.0 (2)
P1—O1—C1—C6	100.9 (2)	P1—N2—C14—C19	−30.3 (3)
P1—O1—C1—C2	−83.8 (2)	P1—N2—C14—C15	149.3 (2)
C6—C1—C2—C3	−1.6 (4)	C19—C14—C15—C16	−0.3 (4)
O1—C1—C2—C3	−176.8 (2)	N2—C14—C15—C16	−179.9 (2)
C1—C2—C3—C4	1.4 (4)	C14—C15—C16—C17	2.2 (4)
C2—C3—C4—C5	−0.7 (4)	C15—C16—C17—C18	−2.6 (4)
C3—C4—C5—C6	0.1 (4)	C15—C16—C17—C20	179.6 (2)
C2—C1—C6—C5	1.1 (4)	C16—C17—C18—C19	1.1 (3)
O1—C1—C6—C5	176.3 (2)	C20—C17—C18—C19	178.9 (2)
C4—C5—C6—C1	−0.3 (4)	C15—C14—C19—C18	−1.2 (3)
P1—N1—C7—C8	94.9 (3)	N2—C14—C19—C18	178.4 (2)
N1—C7—C8—C9	−140.9 (3)	C17—C18—C19—C14	0.8 (3)

### Hydrogen-bond geometry (Å, °)

**Table:**

*D*—H···*A*	*D*—H	H···*A*	*D*···*A*	*D*—H···*A*
N1—H1N···O1^i^	0.76 (3)	2.43 (3)	3.127 (3)	153 (3)
N2—H2N···O2^i^	0.86 (3)	1.91 (3)	2.761 (3)	176 (3)

Symmetry codes: (i) *x*+1/2, −*y*+2, *z*.
